# Continuous femoral nerve blockade and single-shot sciatic nerve block promotes better analgesia and lower bleeding for total knee arthroplasty compared to intrathecal morphine: a randomized trial

**DOI:** 10.1186/s12871-017-0355-x

**Published:** 2017-05-12

**Authors:** Nora Elizabeth Rojas Álvarez, Rosemberg Jairo Gomez Ledesma, Adilson Hamaji, Marcelo Waldir Mian Hamaji, Joaquim Edson Vieira

**Affiliations:** 1 Hospital das Clínicas, Divisão de Anestesia, Rua Dr. Ovídio Pires de Campos, 471, Cerqueira César, São Paulo, SP Brazil CEP 05403-010; 2 Institute of Orthopedics and Trauma Surgery, Hospital das Clínicas, Rua Dr. Ovídio Pires de Campos, 333, Cerqueira César, São Paulo, SP Brazil CEP 05403-010; 3 Department of Surgery, University of São Paulo Medical School, Av. Dr. Arnaldo 455, sala 2345, Cerqueira César, São Paulo, SP Brazil CEP 01246-903

**Keywords:** Analgesia, Nerve Block, Pain, Postoperative, Arthroplasty, Replacement, Knee, Anesthesia, Conduction

## Abstract

**Background:**

Knee arthroplasty leads to postoperative pain. This study compares analgesia and postoperative bleeding achieved by intrathecal morphine with a continuous femoral plus single-shot sciatic nerve block.

**Methods:**

A randomized non-blinded clinical trial enrolled patients aged over 18 years old, ASA I to III who underwent total knee arthroplasty. All patients underwent spinal anesthesia with isobaric bupivacaine, 20 mg. One group received 100 mcg of intrathecal morphine (M group), and the other received a femoral nerve block by continuous infusion plus a "single shot" block of the sciatic nerve at the end of the surgery (FI group). Pain score from verbal numeric rating scale (VNRS) and morphine consumption during the first 72 h, as well as motor blockade, adverse effects, and postoperative bleeding were recorded. Analysis of variance of repeated measures with Bonferroni post-test, *t*-test and Fisher exact test were used for statistical analysis.

**Results:**

Thirty nine patients completed the study (M = 20; FI = 19 patients) and were similar except for higher age in the FI group. Motor blockade as well as movement pain during postanesthesia care unit (PACU) staying were not different between the groups, but movement pain was significantly lower in FI group after 24 h. Postoperative bleeding (ml) was lower in FI group.

**Conclusions:**

Continuous femoral nerve block combined with sciatic nerve block provides effective for postoperative analgesia in patients undergoing total knee arthroplasty, with lower pain scores after 24 h and a lower incidence of adverse effects and bleeding compared to intrathecal morphine.

**Trial registration:**

Retrospectively registered on https://clinicaltrials.gov/ under identifier NCT02882152, 23^rd^ December, 2016.

## Background

Knee arthroplasty leads to postoperative pain. Several techniques using regional anesthesia may provide effective analgesia and early recovery, as well as prevent thromboembolic events. A rapid rehabilitation allows higher patient satisfaction and lower costs [1].

Regional anesthesia is superior to general anesthesia with better postoperative pain control without the use of opioids and its adverse effects. For some operations, analgesia can be extended longer into the postoperative period by means of continuous infusions through epidural catheters [2]. In addition, spinal anesthesia plus opioids ensures quite effective analgesia, even though its side effects may delay the rehabilitation process [3, 4].

A meta-analysis of 185 patients suggested equivalent postoperative pain control comparing femoral nerve block and intrathecal morphine with fewer side effects with the femoral nerve blockade [5]. Continuous regional analgesia of the femoral nerve via a catheter may also extend analgesia in line with the patients’ demands [6].

The sciatic nerve block is still controversial when it comes to analgesia for knee arthroplasty because this benefit is obtained at the expense of motor blockade of leg and foot, which functionally is of considerable importance during the immediate postoperative period [7].

This study compares analgesia achieved with intrathecal morphine administration to a continuous femoral block combined with a sciatic nerve block. Incidence of adverse effects, postoperative bleeding, patient’s satisfaction and motor blockade of both techniques were also evaluated.

## Methods

A randomized non-blinded prospective clinical trial conducted at the Institute of Orthopedics and Traumatology surgery, Hospital das Clinicas of University of São Paulo Medical School (IOT-HCFMUSP) in the period between December 2011 and September 2016. HCFMUSP Ethics Research Committee approved this study under number 0257/09, also registered on https://clinicaltrials.gov under identifier NCT02882152. Written informed consent to participate were obtained from all the participants.

After the signing of the informed consent, forty patients aged over 18 years old, ASA physical status from I to III (American Society of Anesthesiologists) who underwent total knee arthroplasty (TKA) were included and allocated by means of a random number table. Patients aged below 18 years old, with ASA IV or V physical status, infection near the puncture site, coagulation disorders, preexisting neurological disorders, allergy to local anesthetics, pregnancy and lactation, that refused spinal block or to sign an informed consent form were excluded from the study.

Patients were monitored with electrocardiography, noninvasive blood pressure and pulse oximetry. Hydration and type of fluid were at the discretion of the anesthesiologist. Sedation was performed with intravenous (IV) midazolam titrated doses (2–5 mg) and fentanyl (50–100 mcg). All patients underwent spinal anesthesia with 20 mg of isobaric bupivacaine (Cristalia, São Paulo, Brazil). The patients were allocated into two groups: to receive 100 mcg of intrathecal morphine (Cristalia, São Paulo, Brazil) (M group, *n* = 20) or a femoral nerve block with insertion of a catheter for continuous infusion combined with a sciatic nerve block at the end of the surgery (FI group, *n* = 20).

The femoral block used an 'out-of-plane' approach at the level of the inguinal ligament guided by ultrasound (FUJIFILM SonoSite, Bothell, United States) and by neurostimulation (BBraun, São Paulo, Brazil) to localize the nerve. Quadriceps contraction was obtained with an initial stimulation of 1 mA and then disappeared below a current level of 0.3 mA. The needle's position was considered therefore adequate. After negative aspiration, 15 mL of ropivacaine at 0.5% Cristalia, São Paulo, Brazil) was injected. Ultrasound confirmed the dispersion of the solution within the iliac compartment. A catheter was inserted 5 cm beyond the tip of the needle without resistance. A continuous infusion was started using an elastomeric pump (B. Braun, easy pump, São Paulo, Brazil) filled with ropivacaine 0.2% at a rate of 5 ml/h for 48 h. The Raj posterior approach directed the sciatic nerve block guided by ultrasound and neurostimulation until motor response with a current of 0.3 mA. Following negative blood aspiration, a single injection of 15 ml of 0.5% ropivacaine was given [8].

All patients received intraoperatively 2 g of dipyrone and 100 mg of ketoprofen IV for analgesia and antiemetic prophylaxis with 8 mg of ondansetron. Dipyrone 2 g every 6 hours and ketoprofen 100 mg every 12 h were prescribed for postoperative pain at the anesthesiologists' discretion. Tramadol 100 mg IV every 8 hours was offered as a rescue analgesia. If pain persisted with verbal numeric rating scale (VNRS) higher than 3 an infusion of morphine IV 2 mg every 2 h was offered to lower VNRS < 3.

Pain score from VNRS and morphine consumption during the first 72 h after surgery, as well as motor blockade, adverse effects, postoperative bleeding and patient satisfaction were all registered.

Evaluations were performed at post-anesthesia care unit (PACU) discharge and every 24 h up to 72 h after surgery. Pain intensity was evaluated by VNRS (zero = no pain, 1–3 = mild pain, 4–5 = moderate pain, 7–9 = severe pain, 10 = unbearable pain). The degree of patient satisfaction was qualified by a categorical scale (1 = poor, 2 = fair, 3 = good; 4 = excellent). The degree of motor block was assessed on a Bromage scale (zero = no motor block, 1 = can move the knee or foot, but cannot lift the leg, 2 = can only move the foot, 3 = cannot move the knee or foot). Adverse reactions such as nausea, vomiting, urinary retention, dyspnea and itching were registered.

The sample size was calculated to observe an effect size reduction of 25% for VNRS considering two groups and four moments (PACU, and 24, 48 and 72 h), resulting in a total of 30 patients plus a dropout of up to 20% (*n* = 36), at a significance level of 5% and power of 90% [G*Power version 3.1.9.2. http://www.gpower.hhu.de/en.html]. Demographic and monitoring data were compared with *t*-test or Fisher exact test. Morphine consumption, VNRS and Bromage results were compared by analysis of variance (ANOVA) of repeated measures followed by Bonferroni test. A significance level of 5% was considered significant for a statistics power of 90%.

## Results

**Fig. 1:**
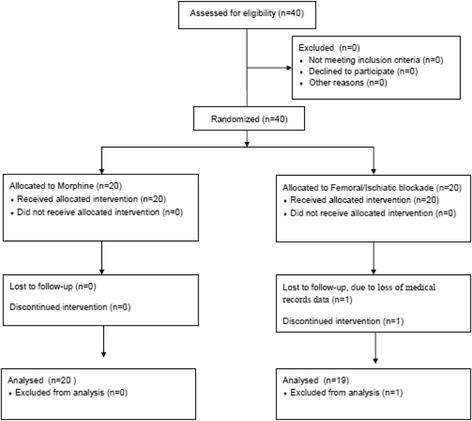
Flowchart of eligibility and participation

Thirty-nine patients completed the study (one dropout due to loss of medical records data in FI group; Fig. 1). The groups were similar except for age, higher in the FI group (Table 1).

**Table 1:** Demographic and baseline values

	Groups		*p*-value
	Intratechal morphine	Femoral blockade	
	M (*n* = 20)	FI (*n* = 19)	
Age	58.3 ± 10.5	67.3 ± 8.3	<0.01
Sex (F/M)	14/6	16/3	0.45
Weight	77.5 ± 16.9	70.7 ± 20.0	0.25
Systolic BP	132.1 ± 20.0	136.2 ± 22.9	0.58
Diastolic BP	76.2 ± 13.5	72.1 ± 21.1	0.5
HR	72.5 ± 9.4	76.4 ± 9.1	0.2
SatO2	96.3 ± 1.5	96.1 ± 1.4	0.62

*BP* Non-invasive Blood pressure, *HR* heart rate, *SatO2* oxygen saturation from pulse oximetry. All analysis using *t* test (*significant), except for sex compared by means of Fisher exact test

There was no difference between the groups for pain in movement during PACU staying. After PACU discharge, pain intensity was significantly lower in the FI group (Table 2). Twenty percent of patients in M group required rescue analgesia with tramadol and 10% with morphine.

**Table 2:** Assessment of pain during movement while in PACU, VNRS

	Groups (*n*)		Mean difference	*p*-value
	Intratechal morphine	Femoral blockade		
	M (*n* = 20)	FI (*n* = 19)		
PACU (zero)	0.1 ± 0.1	0.0 ± 0.0	0.1 ± 0.1	0.30
24 h	3.0 ± 0.5	0.6 ± 0.2	2.3 ± 0.5	<0.01
48 h	3.5 ± 0.4	0.3 ± 0.1	3.2 ± 0.4	<0.01
72 h	2.6 ± 0.3	0.1 ± 0.1	2.5 ± 0.3	<0.01

ANOVA of repeated measures followed by Bonferroni test

Postoperative bleeding volume (ml) was lower in the FI group at all times (Table 3). The motor blockade intensity showed no difference during PACU staying, but at 24 h four patients from the FI group showed a score of 1 and two scores of 2 (Bromage scale); at 48 h no patient had any motor blockade.

**Table 3:** Postoperative bleeding volume in milliliters (ml)

	Groups (n)		Mean Difference	*p*-value
	Intrathecal morphine	Femoral blockade		
	M (*n* = 20)	FI (*n* = 19)		
PACU (zero)	271.8 ± 33.3	136.5 ± 20.5	135.3 ± 39.1	0.001
24 h	334.7 ± 39.6	180.5 ± 19.7	154.2 ± 44.2	<0.01
48 h	111.9 ± 28.4	69.2 ± 20.7	42.6 ± 35.2	<0.01
72 h	155.3 ± 39.1	20.0 ± 0.0	135.3 ± 39.1	<0.01

ANOVA of repeated measures followed by Bonferroni test

A majority of patients from the M group had pruritus as the main complaint in the PACU and in the first 24 h (*n* = 14, 70%), followed by urinary retention (*n* = 10, 50%), but not much nausea (*n* = 5, 25%) and vomiting (*n* = 1, 5%). No neurological complications, hematomas or systemic toxicity of the local anesthetic were observed in the FI group at any time. Patient satisfaction was excellent for FI at a rate of 100%. In M group, 20% were completely satisfied, but an equal number found it a bad experience, mainly due to pain (Table 4).

**Table 4:** Adverse events frequency and satisfaction rate, at PACU and 24 h (h) moments

	Groups				*p*-value
	Intratechal morphine		Femoral blockade		
	M (*n* = 20)		FI (*n* = 19)		
	PACU	24 h	PACU	24 h	
Pruritus	14	7	-	-	<0.01*
Urinary retention	10	7	-	-	<0.01*
Nausea	5	6	-	-	<0.05**
Vomiting	1	3	-	-	>0.05
Satisfaction (%) at 24 h	20		100		<0.05

*Significant for both moments of PACU and 24 h analysis at *p*-values < 0.01

**significant for both moments of PACU and 24 h analysis at *p*-values < 0.05. Fisher exact test

## Discussion

This study showed that continuous femoral block combined with sciatic nerve block provides better postoperative analgesia than intrathecal morphine for total knee arthroplasty.

In previous studies, intrathecal morphine has been considered more effective, except for pruritus, in comparison with femoral nerve block [9, 10]. A low dose of intrathecal morphine was considered safer than the femoral nerve blockade [11]. This study suggests, however, a better result for a femoral approach that may be due to a prolonged analgesia provided by a continuous blockade.

This continuous nerve block prolonged analgesia, reduced the consumption of rescue opioids as well as promoted early functional activity with no report of related accidental fall. The infusion pattern and location of the catheter were based on our clinical experience, but aimed to reach an analgesic quality under lower risk of nerve lesion [12]. One advantage of the femoral block for this surgery is the relaxing effect of the quadriceps muscles that provide greater tolerance to lower limb passive mobilization [13]. On the other hand, due to this muscle weakness, some authors consider it an independent risk factor for accidental fall in the postoperative period [14, 15]. Adductor canal block has been shown to preserve this muscle strength, but not enough to promote early mobilization and without a significant difference in postoperative pain [16, 17]. More recently, a single-shot femoral blockade combined with low dose spinal anesthesia seemed as an alternative to conventional spinal anesthesia in outpatient arthroscopic meniscus repair [18].

The sciatic nerve block remains controversial [19–21]. An inadequate sciatic nerve block may result in higher morphine consumption [10]. It seems to be useful in lateral arthroplasties due to the participation of some nerve fibers from peroneal innervation in this region [21]. Continuous sciatic nerve block combined with continuous femoral nerve block reduced consumption of opioids, the incidence of nausea and vomiting, and promoted better postoperative pain control in the 36 h immediately after TKA [22]. A systematic review, however, did not support these studies [20]. And femoral and sciatic nerve block promoted shorter hospital staying and superior postoperative pain control up to 12 h compared to femoral block plus local infiltration, even though local infiltration could preserve quadriceps function in the immediate postoperative period as an advantage [23–25].

The tendency to avoid sciatic block may be related to the risk of injury during TKA with an incidence ranging from 0.2 to 2.4%. The main risk factors are valgus deformity, tourniquet's ischemia longer than 120 min, pre-existing neuropathy and intraoperative bleeding [26]. This prolonged motor blockade can also hide an iatrogenic nerve injury [21]. The choice for the sciatic blockade in this study was to promote better analgesia by means of a considerable volume (15 ml) of long duration local anesthetic (ropivacaine). No postsurgical neuropathy was observed.

The lower opioid consumption and consequent adverse effects such as nausea, vomiting, pruritus and urinary retention probably helped for the greater satisfaction, despite the more intense motor blockade. The results agree with a recent meta-analysis where sciatic and femoral blocks significantly reduce postoperative opioid consumption and reduce surgical pain compared to sole femoral nerve block [27]. Notwithstanding, a multimodal analgesia approach may provide the best results, even as further research is still needed [28].

Less postoperative bleeding occurred in the femoral group. Although there is no straightforward explanation, a hypothesis considers that greater motor blockade and better analgesia prompted less postoperative mobilization, as well as a possible vasoconstrictor effect of ropivacaine in continuous infusion [29].

Some limitations need to be considered. The use of a double variable, that is femoral catheter plus a sciatic blockade instead of only a continuous femoral block constrains the understanding that analgesia is mainly provided by a femoral block, and a third group would have addressed this issue. The study lacks blinding, which could have been accomplished by placing a catheter in the femoral nerve with placebo infusion to patients receiving intrathecal morphine, but this would be an increased risk of complications. Also, hospital staying or outcomes from physical therapy were not evaluated to observe the true impact of analgesia on postoperative recovery.

## Conclusions

Continuous femoral nerve block combined with sciatic nerve block provides effective for postoperative analgesia in patients undergoing total knee arthroplasty, with lower pain scores after 24 h and a lower incidence of adverse effects and bleeding compared to intrathecal morphine.

## Abbreviations

ANOVA: Analysis of variance
ASA: American Society of Anesthesiologists
FI: Femoral infusion
HCFMUSP: Hospital das Clinicas of University of São Paulo Medical School
IOT-HCFMUSP: Institute of Orthopedics and Traumatology surgery, Hospital das Clinicas of University of São Paulo Medical School
IV: Intravenous
M: Morphine
PACU: Post-anesthesia care unit
TKA: Total knee arthroplasty
VNRS: Verbal numeric rating scale
